# Bridging the “retrieval gap”: Familiar schema-governed categories support reminding of disparate events

**DOI:** 10.3758/s13421-026-01850-0

**Published:** 2026-03-14

**Authors:** Lucas Raynal, Evelyne Clément, Hippolyte Gros, Emmanuel Sander

**Affiliations:** 1 IDEA, Université de Genève, boulevard du Pont-d’Arve, 1211 Genève, Switzerland; 2 Paragraphe, Université CY Cergy Paris and Université Paris 8, 349 Gennevilliers, EA France; 3 CRPN, CNRS, UMR 7077, Aix Marseille Université, Marseille, France

**Keywords:** Analogy, Memory retrieval, Transfer, Relational categories, Schema-Governed Categories

## Abstract

We investigated the role of surface and structural similarities in the retrieval of everyday life events by means of a novel experimental paradigm in which participants could report any memories that came to mind while reading anecdotes instantiating familiar Schema-Governed Categories (SCGs, e.g., *making up an excuse*). Based on the idea that the availability of memories sharing both surface and structural similarities may conceal participants’ ability to retrieve purely structural matches, we presented participants with short narratives describing associations of surfaces and structures which are either usually encountered (e.g., making up an excuse not to hang out with someone, Experiment 1) or less frequent in everyday life (e.g., making up an excuse not to go to an art exhibition, Experiment 2). The results from Experiment 1 replicated the traditional finding that when they are available, memories combining surface and structural similarities prevail over purely structural matches. However, Experiment 2 revealed that when a more direct competition was set between purely surface and purely structural matches, structurally based retrievals were the most frequent. Experiment 3 showed that target cues deprived of any surface features (e.g., *making up an excuse*) elicited more retrievals of usual than unusual surface/structure combos, suggesting the influence of association frequency in analogical retrieval. The data provide the first line of evidence suggesting that participants rely on highly familiar SGCs to process structural encodings and retrieve past experiences based on structural similarities. We discuss the implications of these findings regarding current theories of analogical retrieval holding that this process is predominantly guided by surface similarities.

## Introduction

Analogical thinking is essential to transfer knowledge from one context to another in a way that promotes understanding of incoming experiences (Chalmers et al., 1992; Day & Goldstone, 2012; Hofstadter & Sander, 2013; Holyoak & Thagard, 1995). Crucially, deep analogies often involve finding abstract commonalities (i.e., structural similarities) between two situations regardless of whether they involve similar content at the most concrete level of description (i.e., surface similarities), such as between two situations where someone “makes up an excuse”, as different as these excuses and their goal might be. A widely held view is that analogies are generally successfully implemented when two representations are held in working memory (Forbus et al., 2017; Gentner, 1983; Hummel & Holyoak, 1997). This is because a mapping process allows people to find structural similarities between situations exhibiting different surface features. Nonetheless, in most cases where analogies can be drawn, a single situation is processed (i.e., the target cue), and the source analog has to be retrieved from past experiences stored in long term memory. Therefore, understanding how frequently analogies are implemented, and thus how central analogy is to our cognition, necessitates determining whether people are capable of retrieving analogs on the basis of structural similarities, or whether they mostly rely on surface similarities to retrieve past experiences.

## The history of the surface superiority account

Since the 1980's, researchers have dedicated a great amount of research to understand the conditions under which analogical retrieval generally occurs (Day & Goldstone, 2012; George & Wiley, 2018; Holyoak, 2012; Trench & Minervino, 2020). Most studies have employed the “two-phase” experimental paradigm (Day & Goldstone, 2012; Hofstadter & Sander, 2013; Olguín et al., 2022), where the researchers manipulate the kinds of similarities shared by two sets of problems or stories introduced to the participants within separated sessions to assess their influence on retrieval (see Table 1 for a sample of stimuli used in these studies). The findings have demonstrated that distant analogs (with structural similarities but no surface similarities) are hardly ever retrieved spontaneously, whereas close analogs (with structural similarities and surface similarities) and even surface matches (with surface similarities but no structural similarities) are readily accessed by participants (Gentner et al., 1993; Gick & Holyoak, 1980, 1983; Keane, 1987; Ross, 1987, 1989). They suggested a gap between the difficulty of retrieving distant analogs and the relative ease of mapping them, which has been termed the “retrieval gap” (Holyoak, 2012). The conclusion that analogical retrieval would be strongly constrained by surface similarities has been generally interpreted as the consequence of situation-specific encodings providing insufficient abstract relational cues between distant analogs (Gentner et al., 2003; Jamrozik & Gentner, 2020).

**Table 1:** Sample of stimuli used in previous studies on analogical retrieval

Gick & Holyoak (1980, after Duncker, 1945). Suppose you are a doctor faced with a patient who has a malignant tumor in his stomach. It is impossible to operate on the patient, but unless the tumor is destroyed the patient will die. There is a kind of ray that can be used to destroy the tumor. If the rays reach the tumor all at once at a sufficiently high intensity, the tumor will be destroyed. Unfortunately, at this intensity the healthy tissue that the rays pass through on the way to the tumor will also be destroyed. At lower intensities the rays are harmless to healthy tissue, but they will not affect the tumor either.
Gentner et al. (1993). Karla, an old hawk, lived at the top of a tall oak tree. One afternoon, she saw a hunter on the ground with a bow and some crude arrows that had no feathers. The hunter took aim and shot at the hawk but missed. Karla knew the hunter wanted her feathers so she glided down to the hunter and offered to give him a few. The hunter was so grateful that he pledged never to shoot at a hawk again. He went off and shot deer instead.
Olguín et al. (2022). I have a friend that made an effort in her studies and consequently she obtained an achievement in something related to her studies: She stayed at home all weekend studying and consequently she obtained excellent marks.

The distant analog corresponding to each stimulus instantiated the same critical structure in the context of a general who could not send his whole army along one path to capture a fortress (Gick & Holyoak, 1980), a country which offered to sell its computers to a warlike neighbor to ensure its safety (Gentner et al., 1993), and making an effort to achieve success in something related to health (Olguín et al., 2022)

A ray of hope is that as domain knowledge increases, experts can rely on relational categories to process structural encodings (Chi et al., 1981; Goldwater & Gentner, 2015; Rottman et al., 2012). Relational categories differ from entity categories in that they cohere based on a core relationship that is fulfilled by all members (Gentner & Kurtz, 2005). Specifically, Schema-Governed Categories (SGCs) are a type of relational category that represent the underlying relational structure of situations as a network connecting typical components, even if these vary across instances (e.g., *trip, visit, robbery*; Goldwater et al., 2011). There is evidence that encoding distant analogs in terms of a shared SGC supports structurally based retrieval in experts (Goldwater et al., 2021; Jamrozik & Gentner, 2020; Novick, 1988). Indeed, applying a common schema across instances helps de-emphasize surface features and highlight structural components, thereby strengthening the match between distant analogs.

The possibility that relational categories enhance structurally-based retrieval is also supported by a great deal of studies showing that abstracting a schema through analogical comparison promotes distant analog retrieval (Catrambone & Holyoak, 1989; Gentner et al., 2009; Kurtz & Loewenstein, 2007). Capitalizing on this work, Kurtz and colleagues proposed that inviting participants to represent a principle underlying a set of examples as a category of situations makes it easier to access while encountering structurally similar cases (the category status hypothesis; Kurtz & Honke, 2020; Snoddy & Kurtz, 2021). Snoddy and Kurtz (2021) provided participants with a category-building task inviting them to learn a novel SGC (*problem-as-a-solution*) based on sequential summarization of several analogous problems associated with a common label and training to apply the category to new instances. They found that this setting was particularly efficient to promote structurally-based retrievals when encountering superficially different problems. This finding illustrates that experimental settings inviting participants to represent situations as instances of a relational category can help overcome the surface superiority in retrieval.

In sum, the conclusion that surface similarities are a main determinant of analogical retrieval has motivated a broad line of research investigating experimental settings allowing participants to overcome this surface bias (Catrambone & Holyoak, 1989; Gentner et al., 2009; Snoddy & Kurtz, 2021). A primary contribution of this work is to empirically demonstrate that learning a new SGC enables structurally-based retrievals that would otherwise typically fail. However, several researchers argue that classical studies have underestimated non-experts’ ability to retrieve distant analogs in naturalistic settings, as they may encode everyday situations more deeply than the experimental stimuli used in two-phase designs (Dunbar, 2001; Day & Goldstone, 2012; Hammond et al., 1991; Popov et al., 2020; Raynal & Sander, 2025).

## Analogies in everyday life

Two factors influencing encoding may explain why structural analogies are more likely to be implemented in everyday life than within classical experiments. First, according to Hofstadter and Sander (2013), structurally-based retrieval is commonplace in everyday life because familiar SGCs (*procrastination, tense atmosphere, pleasant surprise, killing-two-birds-with-one-stone*) can be used to encode the structure of most situations and provide retrieval cues to previous instances (Raynal, 2022; Raynal & Sander, 2025; Sander & Richard, 2005; Schank, 1999). The spontaneous activation of familiar SGCs (e.g., *superstition*) during the processing of everyday situations has been empirically attested by Minervino et al. (2023). Specifically, the authors showed that participants tended to draw inferences that align with the activated category in novel instances − for example, expecting a superstitious act before an exam after observing one before a soccer penalty. Whereas such familiar categories appear to be routinely activated in everyday life, traditional lab settings where the stimuli share particularly unfamiliar SGCs (e.g., *convergence schema, permutations, contingent contract, positive feedback loop*, etc.) may be hardly conducive to structural encodings.

Relatedly, a second line of criticism has focused on the procedure of the two-phase paradigm, in which source and target cue situations are presented in the context of an experiment. Dunbar (2001) proposed that the task of passively reading problems or stories induces a superficial encoding, whereas the production of analogies to achieve personally significant goals in real life may be more conducive to processing structural encodings. To replicate this ecological setting in an experimental context, Blanchette and Dunbar (2000) developed a production paradigm that allows participants to draw on their own analogical sources to achieve a specific goal. The participants were asked to imagine that they had been hired by a non-profit organization to persuade citizens and the government that a particular political strategy was either justified or flawed. The authors found that participants mainly generated analogies from sources outside the political and economic domains. These findings illustrated that a naturalistic encoding context is more conducive to observing structurally-based retrievals than classical experimental conditions (see also Hammond et al., 1991). In sum, a naturalistic encoding context and the possibility to encode events via familiar SGCs are two factors that may explain the greater ease of retrieving distant analogs in everyday life.

## The retrieval of everyday events instantiating familiar categories

Recently, Minervino, Trench, and their colleagues have conducted a series of studies to reassess the role of surface and structural similarities in retrieval (Minervino & Trench, 2024; Olguín et al., 2022; Trench et al., 2025; Trench & Minervino, 2015). Most crucially for the present study, Olguín et al. (2022) assessed whether a situation involving an SGC (*robbery* of a bicycle) was more likely to elicit the retrieval of a specific close analog (e.g., having a vehicle stolen) than a distant analog (e.g., having an electronic password stolen). They found that participants were more likely to retrieve close analogs than distant analogs. In a second experiment, the authors replicated their results while investigating retrieval based on two SGCs linked by a causal relation (e.g., *effort causes achievement*, see Table 1). These findings suggested that analogical retrieval is strongly influenced by surface similarities, even when episodes have been encoded in terms of SGCs. The authors argued that the low retrieval rates of close analogs in Blanchette and Dunbar’s (2000) study can be accounted for by experimental biases such as analogy fabrication and uneven availability of close and distant analogs in memory.

Nevertheless, as acknowledged by the authors, the rare occurrence of structurally-based retrievals in this study does not necessarily mean that structural similarities only have a weak influence on retrieval of everyday events. Notably, Thagard et al. (1990) have outlined that analogical retrieval is a comparative process by which the selection of the source with the greatest overlap tends to suppress the activation of other source candidates.^1^ Thus, the availability of close analogs in participants’ memory may have hindered the retrieval of distant analogs in Olguín et al.’s (2022) study. Considering that close analogs share a combination of surface and structural similarities, it is difficult to conclude which type of similarities is mainly responsible for their high retrieval rate (Raynal et al., 2020; Wharton et al., 1994).

Namely, the activation of an SGC may initiate a search in memory for other, structurally similar, instances within that category, and surface similarities may be used as an additional cue to select a specific instance for retrieval. This view predicts that close analogs are primarily retrieved when they are available, but that distant analogs are preferentially retrieved over surface matches when close analogs are missing. We now introduce the empirical approach adopted to test this hypothesis.

## The present study

What is needed to better understand how surface and structural similarities affect the retrieval of events involving familiar SGCs is to pit the two kinds of similarities against each other to assess their respective role in access (Catrambone, 2002; Holyoak & Koh, 1987; Gentner et al., 1993; Raynal et al., 2020). In the study led by Olguín et al. (2022), the target cues preserved associations between surfaces and structures that participants may have typically encountered in everyday life situations (e.g., it is quite common to hear about efforts leading to achievements in a school context). Thus, the surface and structural cues in the experimental stimuli converged to activate the same events in participants’ memory.

However, one way of disentangling the role of surface and structural similarities in driving the retrieval of everyday life events is to present participants with target cues dissociating the combinations of surfaces and structures which are usually found in everyday life. For instance, the familiar structure *pretending to like a gift* may typically involve a *cloth* as a gift, but it may less frequently deal with a *school supply*. These unusual surface/structure combos (e.g., “I don’t like the pen he gave me for university but I preferred not to tell him”) create a more direct retrieval competition between surface matches and distant analogs. In the current paper, we presented participants with these types of target cues to assess our central claim that the retrieval of everyday events instantiating a familiar SGC is mostly driven by structural similarities. Specifically, we predicted that such target cues will elicit more retrievals of distant analogs than surface matches.

We devised a free-reminding experimental paradigm in order to investigate the type of similarities driving the spontaneous retrieval of past events instantiating familiar SGCs. In this paradigm, participants are presented with anecdotes as target cues and are tasked with reporting any retrieval that would have occurred to them during their reading. Thus, our design contrasted with the one used by Olguín et al. (2022) in that participants were allowed to retrieve several memories and we did not specifically focus on the retrieval of a single distant analog for each target cue. In line with Gentner et al. (2009), we collected all the memories that participants retrieved, and subsequently categorized them according to their degree of surface and structural similarities with the corresponding target cue. It allowed us to document the properties of all the memories spontaneously recalled by the participants instead of speculating why certain specific memories of interest were not retrieved.

Due to these differences between the experimental design used in Olguín et al. (2022) and the current study, a first experiment was conducted to ensure that the dominance of close analogs over distant analogs would also be found in a free-reminding task, as long as participants are presented with usual surface/structure combos (Experiment 1). This prediction is motivated by the idea that, whether surface or structural similarities prevail in retrieval, combining them should lead to maximizing retrieval probabilities. The critical experimental manipulation aimed at opposing surface and structural similarities was introduced in Experiment 2, where we varied the surface features of the target cues from Experiment 1 to create unusual surface/structure combos. This specific variation between these two experiments was intended to ensure that any potential differences in results would be due to the critical experimental manipulation (i.e., the usualness of the surface/structure associations) rather than to any other aspects of the experimental design. In Experiment 2, we tested our central prediction that distant analogs outcompete surface matches in retrieval. Lastly, Experiment 3 was aimed at investigating additional factors guiding the selection of an analog during retrieval.

## Experiment 1

### Method

#### Participants

Participants (*N* = 133, 110 females, *M* = 23.1 years, *SD* = 5.3 years) were French-speaking students from the University of Geneva who received course credits for their participation. An additional participant was tested but excluded from analyses for failing to respond to any of the experiment’s questions. Note that participants who provided an answer (being “yes” or “no” to the question regarding the occurrence of a retrieval, cf., the next section) to only one or a few target cues were included in the analyses (their lack of answer was considered as a non-retrieval). Participations were solicited during a class session, and a link to the Qualtrics-implemented experiment was subsequently sent to the participants. Participants gave their written informed consent according to guidelines of the Declaration of Helsinki.

#### Materials and procedure

When they started the online experiment, participants were first presented with the instructions. They were told that they will have to read five sentences describing various situations and to indicate each time whether the situations brought to mind a memory. The instructions also explicitly stated that the task consisted of recalling existing memories rather than making up situations. After reading the instructions, participants were presented with the first target cue and an icon on which they could click to move on to the next screen. Then, the target cue disappeared, and participants were asked whether the situation had brought to mind a memory. They could choose either “yes” or “no” on the screen. If the answer was “yes,” an answer space appeared on the screen so that they could write down the description while providing as many details as possible. Afterward, participants were asked whether the target cue had reminded them of another memory following the same procedure. If participants indicated that the target cue did not remind them of any memory (or any other memory), the next target cue was introduced. In total, participants could report a maximum of three memories per target cue. This number was set because we reasoned that it was unlikely that a target cue would spontaneously elicit the retrieval of more than three memories. In line, even participants who had to report a maximum of memories in a previous pilot study retrieved 3.4 memories on average (Raynal et al., 2018).

The five target cues involved surface features that are typically preserved by instances of the critical SGC (Table 2). Specifically, the target cue referring to the SGC *being pleasantly surprised* was about one’s performance in an oral presentation at school. The target cue instantiating *giving up on a resolution* was dealing with practicing sport on a regular basis. The target cue instantiating the concept *pretending to like a gift* involved a cloth as a gift. The target cue involving the SGC *adopting a superstitious habit* was about optimizing the chances of getting a good grade at the University. Lastly, the target cue referring to the SGC *making up an excuse* involved declining an invitation to go for a coffee with someone.

**Table 2:** Target cues and coding scheme used in Experiment 1

Target cue situations	Full structural similarities	Partial structural similarities	Surface (domain) similarities
*Being pleasantly surprised* I was convinced that I failed my oral presentation but the teacher’s comments were very positive.	Expecting failure but succeeding at something	Succeeding OR failing at something	Presentation/teacher’s comments (educational outcomes)
*Giving up on a good resolution* I promised myself to do a little bit of fitness every day, so I started to go to the gym, but I dropped out three days later.	Making a resolution but giving up on it	Making a resolution OR giving up on something	Fitness/gym (sports)
*Pretending to like a gift * I didn’t like the sweater he gave me for this winter but I preferred not to tell him.	Keeping one’s real opinion to oneself about someone’s well-intended action	Keeping one’s real opinion to oneself OR performing a well-intended action	Sweater/winter (warm clothes)
*Adopting a superstitious habit* I always take with me my old pencil case because I believe it helps me pass my exams	Turning a superstitious behavior into a habit	Acting on a superstition OR turning something into a habit	Pencil case/exams (academic assessment)
*Making up an excuse* I thought up of telling a friend that I already had plans to go for a coffee with someone else after he asked me to have one with him.	Lying to decline an invitation	Lying OR being invited by someone	Friend/coffee (socialization)

Participants were presented with the target cue situations without the category labels in italics (e.g., *Being pleasantly surprised*)

#### Scoring

Gentner et al.’s (2009) coding scheme was used to classify participants’ retrievals according to the presence or absence of surface and structural similarities (Table 2). The degree to which the reported memory contained the critical concept was rated on a three-point scale (0 = no structural elements, 1 = some structural elements, and 2 = all structural elements). Retrievals were considered as exhibiting no structural similarities when they did not share any abstract component of the SGC characterizing the target cue (structural score of 0). They were categorized as retaining a partial structure when they shared a relational category pertaining to a single relation with the target cue (structural score of 1, e.g., *making a resolution* or *giving up on something* for the target cue *giving up on a resolution*), but not the two crucial segments defining the whole structure. Lastly, reminders were considered as sharing the full structure of the target cue if they shared the two crucial abstract components of the structure (structural score of 2, e.g., *making a resolution* but *giving up on it*). Note that this coding procedure is conservative in determining whether a reported memory can be considered an analog (i.e., with full structural similarities). Consequently, some memories that are actual analogs might not be coded as such due to insufficient details provided by the participants. However, the data from a pilot experiment have suggested that participants generally provide sufficient information to allow the identification of the structure underlying their retrievals (Raynal et al., 2018).

Memories were also categorized as superficially similar or dissimilar according to the presence or absence of at least one semantically close object between the source memory and the target cue (Gentner et al., 2009). As an illustration, reminders were considered as superficially similar to the target cue referring to someone making up an excuse to decline a friend’s invitation to go for a coffee, if it referred to *friend*, *coffee*, or if the situation was more broadly related to the semantic domain *socialization*. Memories were classified into one of six categories by crossing the level of surface similarities (two levels) and structural similarities (three levels).^2^ The hypothesis underlying Experiment 1 was tested by comparing the counts of close analogs (full structural similarities and surface similarities) and distant analogs (full structural similarities and no surface similarities) that were retrieved by each participant.

#### Sample size

A power analysis was performed on G*Power 3.1.9.7. (Faul et al., 2007) to approximate the sample size needed to detect significant differences between the number of each type of retrieval for each target cue. The required number of responses for each target cue was calculated based on an effect size of φ = 0.61 obtained by Olguín et al. (2022) when comparing the number of close and distant analogs that participants retrieved from their own experiences. Sixty responses per target cue were needed for a power of > 0.90 to detect an effect with an alpha level of .01. We targeted the number of participants that was approximately twice the number of responses required according to this analysis as we expected that a substantial proportion of participants would answer “no” to our spontaneous retrieval instruction for some of the presented target cues. We left the online experiment open for 2 weeks in order to recruit approximately 120 participants, which led us to gather data from an effective sample of 133 participants.

### Results and discussion

**Fig. 1:**
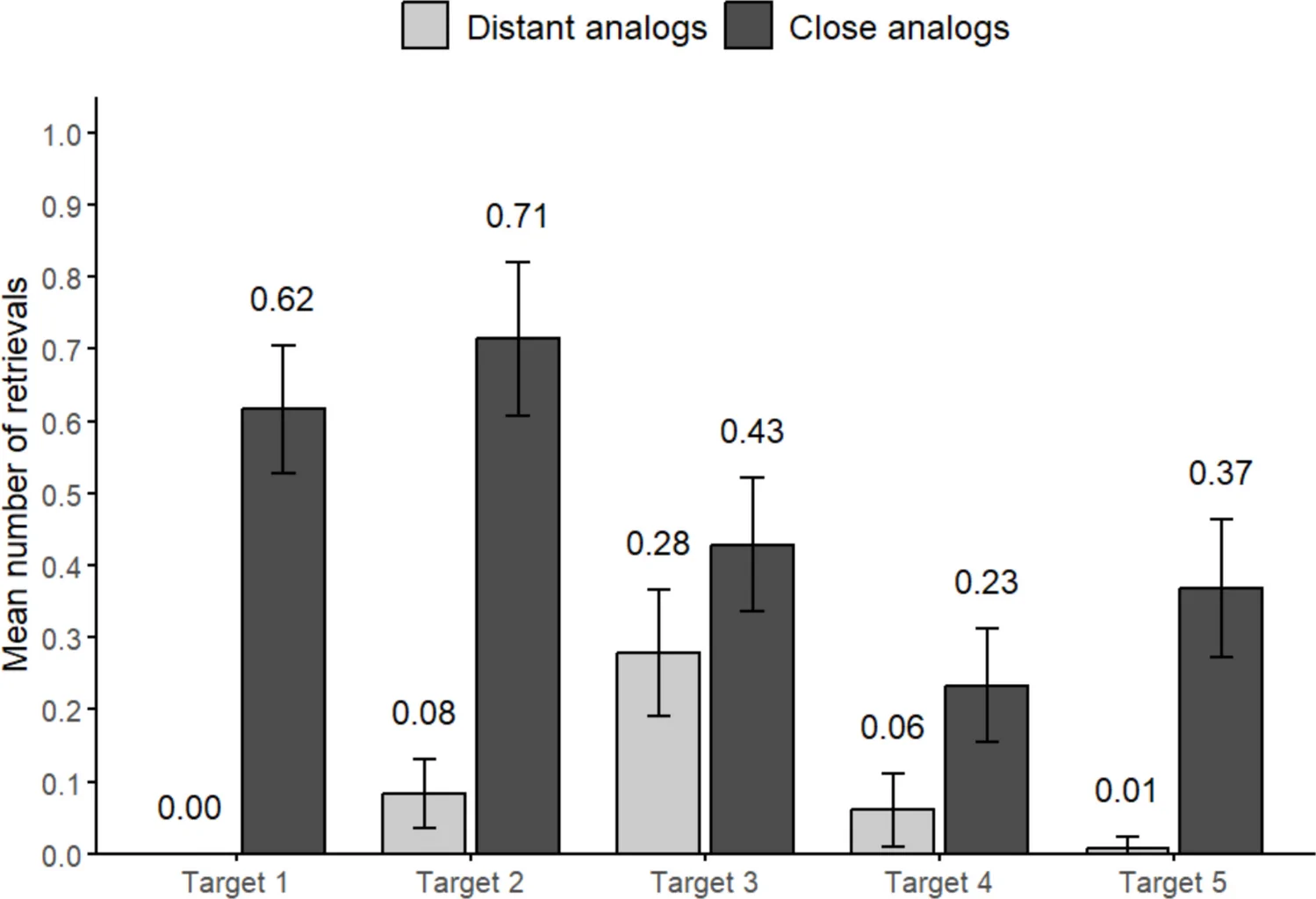
Mean number of retrievals per participant for distant analogs and close analogs (Experiment 1). Error bars represent 95% confidence intervals. Target 1 = *being pleasantly surprised*, Target 2 = *giving up on a resolution*, Target 3 = *pretending to like a gift*, Target 4 = *adopting a superstitious habit*, Target 5 =* making up an excuse*

A total of 502 memories were collected (mean word count per retrieval = 44.82, *SD* = 20.83), for an average of 0.75 reminders per participant for each target cue. The retrieval rates of each type of memory among all the retrievals reported by all participants are displayed in Table 3. A related-samples *t*-test on participants’ mean number of retrievals showed that close analogs (*M* = 0.47, *SD* = 0.57) were retrieved significantly more often than distant analogs (*M* = 0.09, *SD* = 0.31), *t*(132) = 14.45, *p* <.001, Cohen’s *d* = 0.84 (see Fig. 1). This dominance of close analogs over distant analogs was significant for the target cue involving *being pleasantly surprised* (*M* = 0.62, *SD* = 0.52 vs. *M* = 0.00, *SD* = 0.00, *t*(132) = 13.72, *p* <.001, Cohen’s *d* = 1.68), *giving up on a resolution* (*M* = 0.71, *SD* = 0.62 vs. *M* = 0.08, *SD* = 0.28, *t*(132) = 10.23, *p* <.001, Cohen’s *d* = 1.33), *pretending to like a gift* (*M* = 0.43, *SD* = 0.54 vs. *M* = 0.28, *SD* = 0.51, *t*(132) = 2.00, *p* <.05, Cohen’s *d* = 0.29), *adopting a superstitious habit* (*M* = 0.23, *SD* = 0.46 vs. *M* = 0.06, *SD* = 0.30,* t*(132) = 3.49, *p* <.001, Cohen’s *d* = 0.45), and *making up an excuse* (*M* = 0.37, *SD* = 0.56 vs. *M* = 0.01, *SD* = 0.09, *t*(132) = 7.32, *p* <.001, Cohen’s *d* = 0.92).

**Table 3:** Total number of recalls across participants (Experiment 1)

	Full structure	Partial structure	No structure	Total
Surface	319 (63.55%)	96 (19.12%)	15 (2.99%)	430 (85.66%)
No surface	59 (11.75%)	12 (2.39%)	1 (0.20%)	72 (14.34%)
Total	378 (75.30%)	108 (21.51%)	16 (3.19%)	502 (100%)

The dominance of close analogs over distant analogs when the target cues preserve usual surface/structure associations confirms that the combination of surface and structural similarities is particularly effective in driving retrieval. Most importantly, the retrieval rates of close analogs and distant analogs obtained in Experiment 1 were remarkably similar to those obtained by Olguín et al. (2022). This confirms that the two experimental designs are comparable. In Experiment 2, we slightly modified our target cues so that they displayed unusual surface/structure associations, thus creating a more direct retrieval competition between distant analogs and surface matches. This experimental setting allowed us to test our central hypothesis that structural similarities overcome surface similarities in the retrieval of everyday life events instantiating familiar SGCs.

## Experiment 2

### Method

#### Participants

Participants (*N* = 81, 61 females, *M* = 24.2 years, *SD* = 4. 9 years) were French-speaking students from the University of Geneva who received course credits for their participation. An additional nine participants were tested but excluded from analyses since they failed to respond to any question during the experiment. The participation of the subjects was solicited during a class session before a link to the Qualtrics-implemented experiment was sent to them. Participants gave their written informed consent according to guidelines of the Declaration of Helsinki.

#### Materials and procedure

The surface features of the target cues from Experiment 1 were replaced so as to dissociate usual surface/structure combos (Table 4). Both the surface and the structure of the target cues refer to features commonly encountered in everyday life, but their association within a single situation is less frequent than in Experiment 1. The target cue referring to *being pleasantly surprised* was now dealing with the positive criticisms one had received about her theater play rather than about a presentation at school. The target cue instantiating the SGC *giving up on a resolution* became about knitting a little bit everyday instead of doing fitness. Similarly, the target cue instantiating *pretending to like a gift* involved a pen as a gift rather than a sweater. The target cue referring to *adopting a superstitious habit* involved optimizing chances to make a safe boat trip instead of getting a good grade at university. Lastly, the target cue characterized by the SGC *making up an excuse* was about declining an invitation to go to an exhibition rather than a proposition to go for a coffee. The instructions and procedure were identical to those used in Experiment 1.

**Table 4:** Target cues used in Experiment 2

*Being pleasantly surprised* I was convinced that I performed poorly during my theater play but the spectators’ criticisms were very good.
*Giving up on a resolution* I promised myself to knit a little bit every day, so I started knitting a garment, but I gave up three days later.
*Pretending to like a gift* I didn’t like the pen he gave me for university but I preferred not to tell him.
*Adopting a superstitious habit* I always take seaweed with me because I believe it keeps the problems away during my boat trips.
*Making up an excuse* I thought up of telling the painter that I already had to go to another exhibition after he asked me to come and see his own.

Participants were presented with the target cue situations without the category labels in italics (e.g., *being pleasantly surprised*)

#### Frequency of association between surface and structure

A separate task with different participants (*N* = 90) was used to verify that the associations of surface and structural features within the target cues from Experiment 2 were less frequent than those introduced in the target cues from Experiment 1. Participants were presented with five pairs of statements, each referring to a target cue from Experiment 1 and the corresponding target cue from Experiment 2 (sharing the structure but exhibiting a different surface as the target cue from Experiment 1). The task of the participants was to indicate which of the two situations they had most often encountered, whether it had occurred to themselves or to someone else. Participants could also indicate that neither of the two situations had been encountered more frequently than the other. The statements explicitly described both the surface and the structure of the target cues to optimize the encoding of the structure and allow participants to focus on whether the associations between surface and structure were frequent or not. For instance, they were asked to indicate whether they had most often encountered the situation of “pretending to like a pullover that was given to them as a gift” or “pretending to like a pen that was given to them as a gift.” As expected, participants indicated that they had more frequently encountered the target cues used in Experiment 1 than the corresponding target cues from Experiment 2 for the SGC *being pleasantly surprised* (χ^2^ (1, *N* = 79) = 71.203, *p* <.001, *ϕ* = 0.95), *giving up a resolution* (χ^2^ (1, *N* = 75) = 56.33, *p* <.001, *ϕ* = 0.84), *pretending to like a gift* (χ^2^ (1, *N* = 84) = 80.05, *p* <.001, *ϕ* = 0.98), *adopting a superstitious habit* (χ^2^ (1, *N* = 65) = 42.22, *p* <.001, *ϕ* = 0.52), and *making up an excuse* (χ^2^ (1, *N* = 82) = 53.12, *p* <.001, *ϕ* = 0.68). Thus, the associations of surface and structural features within the target cues were less frequent in Experiment 2 than in Experiment 1.

#### Scoring

Two independent raters used the coding scheme from Experiment 1 to categorize memories as belonging to one of six types (i.e., crossing the three levels of structural similarities and the two levels of surface similarities). The structural similarities were rated in an identical way to that in Experiment 1. The coding scheme for surface similarities was adapted to the new surface features of the target cues used in Experiment 2. Retrievals elicited by each of the five target cues presented in Table 4 were considered as superficially similar if they were related to, respectively, *theater play/spectators’ criticisms* (or more broadly to *performing arts*), *knitting/garment* (*handicraft*), *pen/university* (*school supplies*), *seaweed/boat trip* (*transports*), *painter/exhibition* (*visual arts*). This coding scheme allowed us to test our primary hypothesis by comparing the number of distant analogs and surface matches (surface similarities and no structural similarities as defined by our coding scheme) retrieved by each participant.

#### Sample size

A similar rationale to that in Experiment 1 was used to determine the targeted sample size of Experiment 2. In order to attain a minimum of 60 responses by target cues given that, according to the data from Experiment 1, participants would retrieve 0.75 memory for each target cue, we targeted a sample size of approximately 80 participants. In order to attain approximately 80 participants in our final sample, we allowed students to accomplish the experiment over a week, which led to us having an effective sample size of 90 participants.

### Results and discussion

**Fig. 2:**
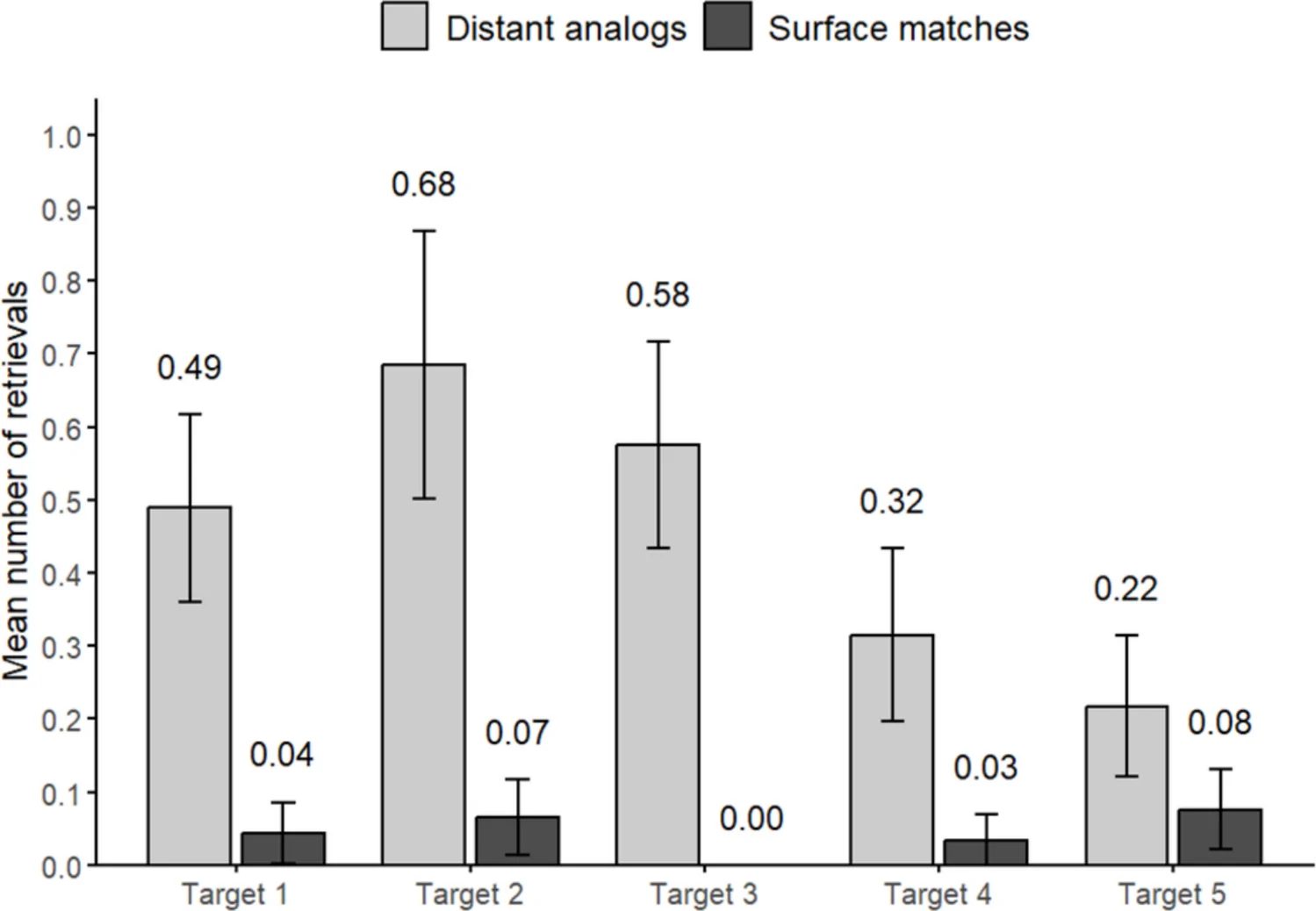
Mean number of retrievals per participant for distant analogs and surface matches (Experiment 2). Error bars represent 95% confidence intervals

A total of 311 memories were collected (mean word count per retrieval = 35.31, *SD* = 15.48), and each participant reported 0.68 memory on average for each target cue. The two independent raters initially agreed on 94.26% of the memories and resolved disagreements through discussion. The total retrieval rates of each type of memory reported by the participants are displayed in Table 5. In line with our main hypothesis, the mean number of retrievals per participant was higher for distant analogs (*M* = 0.46, *SD* = 0.68) than for surface matches (*M* = 0.04, *SD* = 0.20) (see Fig. 2). A related-samples *t*-test revealed that this difference was significant (*t*(80) = 12.06, *p* <.001, Cohen’s *d* = 0.82). The superiority of distant analogs over surface matches was observed for the target cue situation involving *being pleasantly surprised* (*M* = 0.49, *SD* = 0.62 vs. *M* = 0.04, *SD* = 0.21, *t*(80) = 6.39, *p* <.01, Cohen’s *d* = 0.97*), giving up on a resolution* (*M* = 0.68, *SD* = 0.89 vs. *M* = 0.07, *SD* = 0.25, *t*(80) = 6.12, *p* <.01*,* Cohen’s *d* = 0.95), *pretending to like a gift* (*M* = 0.58, *SD* = 0.68 vs. *M* = 0.00, *SD* = 0.00, *t*(80) = 8.09, *p* <.01, Cohen’s *d* = 1.19), *adopting a superstitious habit* (*M* = 0.32, *SD* = 0.57 vs. *M* = 0.03, *SD* = 0.18, *t*(80) = 4.39, *p* <.01, Cohen’s *d* = 0.67), and *making up an excuse* (*M* = 0.22, *SD* = 0.46 vs. *M* = 0.08, *SD* = 0.27,* t*(80) = 2.39, *p* <.05, Cohen’s *d* = 0.37).

**Table 5:** Total number of recalls across participants (Experiment 2)

	Full structure	Partial structure	No structure	Total
Surface	24 (6.91%)	11 (3.03%)	16 (6.30%)	51 (16.40%)
No surface	211 (66.75%)	46 (15.84%)	3 (1.16%)	260 (83.60%)
Total	235 (75.56%)	57 (18.33%)	19 (6.11%)	311 (100%)

By using target cues involving unusual surface/structure combos, we showed that participants rely on familiar SGCs to prioritize structural similarities during retrieval, thereby confirming our main prediction. While SGCs enable participants to proficiently retrieve distant analogs, other factors may have an additional influence on the selection of specific instances from the critical categories. One such factor may be related to the frequency of association between surface and structure in memory: the more instances that share a given surface, the more likely it is that one of these instances will be retrieved (Trench et al., 2009). Furthermore, the secondary role of surface similarities may also lead to a preference for superficially similar instances (i.e., close analogs) over superficially different ones (i.e., distant analogs; Olguín et al., 2022).

Additional analyses were conducted to examine the influence of these two factors on retrieval. The coding scheme used to identify superficially similar memories in Experiment 1 and Experiment 2 was applied by a single rater to determine whether each source analog involved a usual or unusual semantic domain for the critical SGC across the two experiments. For instance, retrieved analogs for the target cues *pretending to like a gift* were coded as related to *clothes* (usual association) or *school supplies* (less usual association). A related-samples *t*-test revealed that usual surface/structure combos (*M* = 0.25, *SD* = 0.46) were retrieved more frequently than less usual ones in Experiment 2 (*M* = 0.06, *SD* = 0.24, *t*(80) = 7.86, *p* <.001, Cohen’s *d* = 0.37), supporting the role of association frequency in retrieval. Then, an independent-samples *t*-test allowed us to show that retrievals of usual surface/structure combos were more frequent when they were cued by superficially similar target cues (in Experiment 1, *M* = 0.47, *SD* = 0.57) than when the target cues were superficially dissimilar (in Experiment 2, *M* = 0.25, *SD* = 0.46, *t*(212) = 7.03, *p* <.001, Cohen’s* d* = 0.41). This analysis is consistent with the idea that surface similarities play a role in the selection of a specific source analog during retrieval. However, it may not lead to definitive conclusions as special caution is required while comparing results across experiments (but note that the experimental context and participants’ characteristics in the two experiments are highly similar). In addition, because the target cues from Experiment 1 and Experiment 2 involved surface features − that could either promote or hinder the retrieval of usual or less usual combos − these exploratory analyses do not allow for a perfect disentanglement of association frequency and surface similarities.

## Experiment 3

In this experiment the effect of surface similarities was neutralized by cueing participants’ retrievals with target cues deprived of any surface features (e.g., *pretending to like a gift*). The effect of association frequency should be observed through a prevalence of usual over less usual combos among participants’ retrievals. Again, we conducted an exploratory analysis of the influence of surface similarities through cross-experiment comparisons, as this effect might be reflected in fewer retrievals of usual combos in Experiment 3 than in Experiment 1, where they were cued by the surface of the target cues.

### Method

#### Participants

Participants (*N* = 95, 71 females, *M* = 24.5 years, *SD* = 3.52 years) were French-speaking students from the University of Geneva who received course credit for their participation. Their participation was solicited during a class session, after which they were sent a link to the experiment hosted on Qualtrics. Participants gave written informed consent in accordance with the guidelines of the Declaration of Helsinki.

#### Materials and procedure

The materials and procedure were identical to those used in the previous two experiments, except that the five target cue situations were replaced by the category labels (*being pleasantly surprised*, *giving up on a resolution*, *pretending to like a gift*, *adopting a superstitious habit*, and *making up an excuse*). Thus, participants read each category description, had to indicate whether it reminded them of a memory and, if so, were asked to report that memory in detail (again, they could report a maximum of three memories).

#### Scoring

The coding scheme developed and validated in Experiments 1 and 2 was used by a single rater to determine the degree of structural similarities and the semantic domain of the retrievals. This coding scheme allowed us to compare the count of retrieved analogs involving the usual versus the less usual surface/structure combo for each participant. For instance, we were able to assess whether the target cue situation *giving up on a resolution* more frequently elicited the retrieval of analogs involving *sport* or *handicraft* (see Table 2 for the full list of usual semantic domains, and the section *Scoring *for the less usual semantic domains).

#### Sample size

The same method was used to approximate sample size a priori as in Experiment 2. By allowing students to complete the experiment during a full week, we were able to collect data from 95 participants.

### Results and discussion

**Fig. 3:**
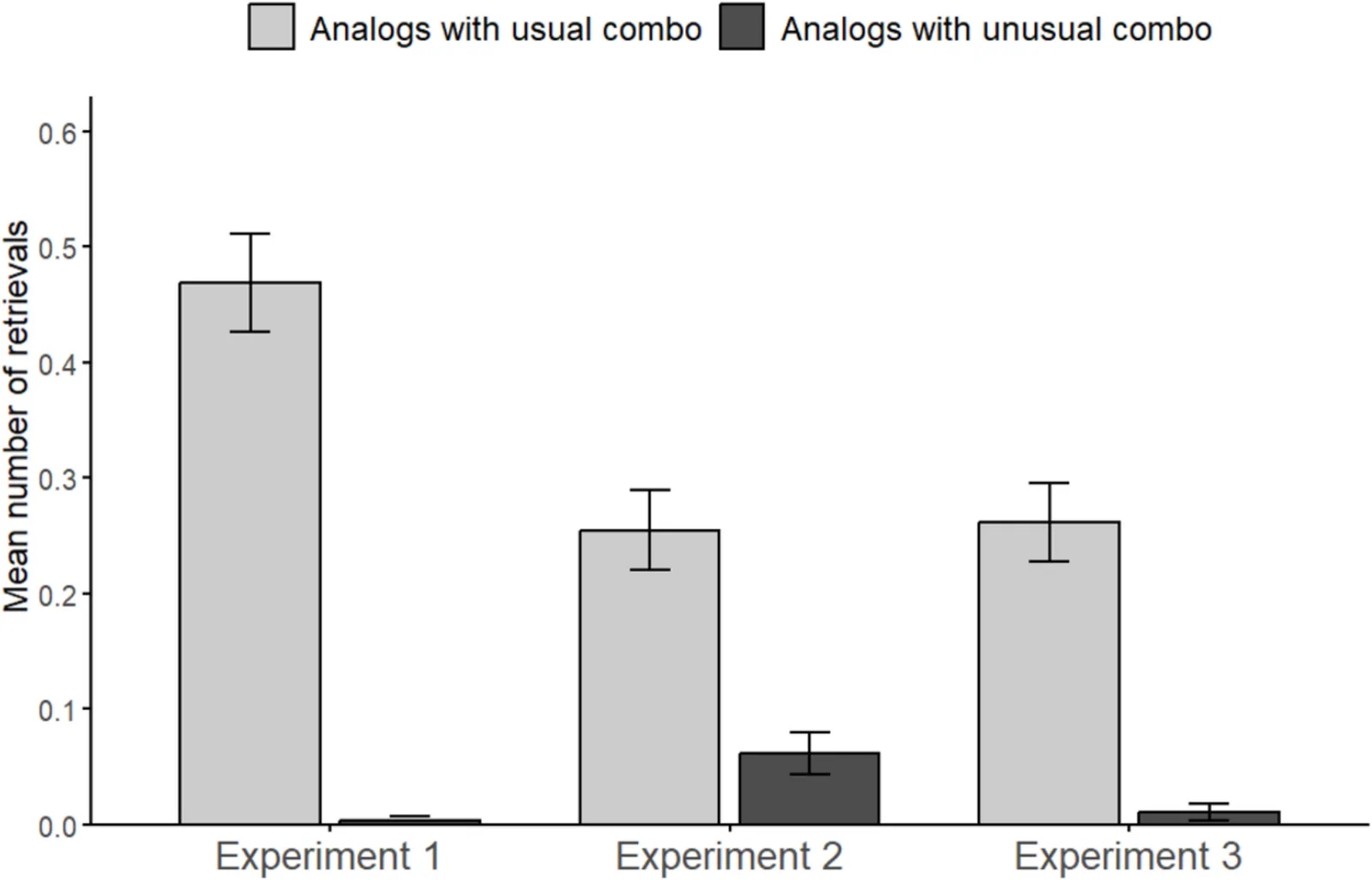
Mean number of retrievals per participant for analogs with usual and unusual surface/structure combos in Experiments 1, 2, and 3. Error bars represent 95% confidence intervals. The analogs with usual surface/structure combos are close analogs in Experiment 1, distant analogs in Experiment 2, and cannot be qualified as close or distant analogs in Experiment 3 where surface features were removed from the target cues

A total of 411 memories were retrieved by the participants (mean word count per retrieval = 35.12, *SD* = 16.41). 338 memories shared full structural similarities with the target cue situation (82.24%), 71 shared partial structural similarities (17.27%), and two did not share any structural similarity (0.49%). The mean number of retrievals for analogs with usual or unusual surface/structure combos within the three experiments is displayed in Fig. 3. In line with the hypothesis that frequency of surface/structure association influences retrieval, a related-samples* t*-test revealed that analogs with usual combos (*M* = 0.26, *SD* = 0.45) significantly outnumbered those with less usual combos among participants’ retrievals (*M* = 0.01, *SD* = 0.10, *t*(94) = 11.82, *p* <.001, Cohen’s *d* = 0.54.). In the context of the exploratory analysis, an independent-samples *t*-test showed that analogs with usual surface/structure combos were retrieved less frequently when faced with the target cues deprived of surface features from Experiment 3 than with the superficially similar target cues from Experiment 1 (*M* = 0.47, *SD* = 0.57, *t*(226) = *−*6.90, *p* <.001, Cohen’s *d* = *−*0.40). These findings support the idea that the frequency of surface/structure associations influence which instances within a SGC are selected during analogical retrieval. They might also suggest that surface similarities influence this process.

## General discussion

The present study aimed to assess the hypothesis that familiar SGCs allow participants to prioritize structural over surface similarities during the retrieval of everyday events. To do so, we designed a free-reminding experimental paradigm in which the retrievals that were spontaneously elicited by anecdotes instantiating a familiar SGC were classified according to the presence of surface and structural similarities. In Experiment 1, we sought to replicate prior findings regarding the dominance of close analogs over other types of matches within the free-reminding experimental paradigm. In line with Olguín et al. (2022), we found that target cues preserving usual surface/structure combos, that is whose surface and structural cues tended to converge towards activating the same memories, mainly elicited the retrieval of close analogs. Based on the idea that the availability of close analogs may conceal participants’ ability to retrieve purely structural matches, we introduced our critical experimental manipulation aimed at creating a more direct retrieval competition between distant analogs and surface matches in Experiment 2. We constructed target cues with unusual surface/structure combos, so that their surface and structural features would tend to cue different events in participants’ memory. The results showed that distant analogs were retrieved dramatically more frequently than surface matches, supporting the prediction that structural similarities outcompete surface similarities in guiding the retrieval of past events involving a familiar SGC. In Experiment 3, we obtained evidence showing that the frequency of surface/structure combos influences the probability of retrieving source analogs. Our exploratory cross-experiment analyses supported the idea that the dominance of close analogs over other types of matches observed in the first experiment was partly due to surface similarities playing a complementary role in analog selection during retrieval. Overall, this study extends previous findings showing the role of SGCs in analogical mapping (Bassok et al., 1995; Minervino et al., 2025), analogical inferences (Minervino et al., 2023), and re-representation (Oberholzer et al., 2018) by illustrating their crucial influence on the stage of analogical retrieval.

### Theoretical implications

The main theories of analogy have long acknowledged that schemas stored in memory can promote structural encodings and that the retrieval of distant analogs fails when these schemas are not available (Catrambone & Holyoak, 1989; Forbus et al., 1995; Gentner et al., 2009; Kurtz & Honke, 2020; Snoddy & Kurtz, 2021). However, the availability of abstract schemas has been considered primarily in expert populations (Goldwater et al., 2021; Jamrozik & Gentner, 2020), while theories advocating surface superiority have largely overlooked non-experts’ reliance on familiar SGCs to retrieve distant analogs. The scarce empirical data gathered to date has not been encouraging regarding the role of familiar SGCs in supporting structurally-based retrieval. Olguín et al. (2022) expressed some deception about the failures to retrieve distant analogs observed in their experiments, despite participants being “proficient users of the exact lexicalized schemas that are needed to uniformly categorize both the base and the target” (p.1411). Our experimental manipulation aimed at pitting surface matches against distant analogs, while limiting opportunities to retrieve close analogs results in a much more positive conclusion. The high rate of distant analog retrievals supports the claim that structurally-based retrievals are commonplace in everyday life.

The accumulating evidence for and against the surface bias might suggest that no coherent conclusion can be drawn regarding the determinants of analogical retrieval. However, Raynal and Sander (2025) have developed a unifying conceptual model of analogical retrieval named ADAPTER (*As Deep As Possible Target Encoding and Retrieval*), where retrieval depends on the deepest encoding supported by one’s available categories. In this model, encoding emphasizes abstract structures when familiar SGCs are available; otherwise, it selects entity categories and focuses on surface features. Encoding context and the salience of structural versus superficial features also shape encoding depth. The authors showed that most empirical findings on analogical retrieval can be accounted for within this coherent framework, including seemingly contradictory findings regarding successes and failures in preferring distant analogs over surface matches. Indeed, ADAPTER accounts for changes in retrieval patterns with increasing expertise (Jamrozik & Gentner, 2020; Goldwater et al., 2021; Novick, 1988). Crucially, it also explains why non-experts are more likely to retrieve distant analogs in everyday life than in traditional experiments due to naturalistic encoding contexts and the availability of familiar SGCs during encoding. The conclusion stemming from ADAPTER is that retrieval is typically guided by surface similarities when unfamiliar stimuli are processed in shallow encoding contexts, but shifts toward structural similarities when everyday situations activate familiar SGCs.

These insights can enrich the computational model MAC/FAC (*Many Are Called, Few Are Chosen*), which accounts for the dominance of surface matches through a two-stage retrieval process (Forbus et al., 1995; see also Hummel & Holyoak, 1997). In this model, MAC selects source candidates based on content similarity with the target, and FAC then applies a structural filter. Surface matches are typically favored over distant analogs because content representations tend to emphasize concrete, situation-specific elements over abstract relational components. In line with the ADAPTER framework, MAC/FAC could incorporate an initial stage in which the encoding of the target situation is shaped by categories available in memory (see Snoddy & Kurtz, 2021, for a similar proposal). This preliminary search could lead to the activation of familiar SGCs or, in their absence, to the recruitment of entity categories only (Raynal & Sander, 2025). Selecting a familiar SGC would enable the construction of a schematized representation through an overarching schema, whereas relying solely on entity categories would yield a more superficial encoding. The resulting representation − whether schematized or superficial − will differ in its degree of content overlap with structurally similar targets, thereby influencing its likelihood of being selected during the MAC stage (Gentner et al., 2009).

According to the category status hypothesis proposed by Snoddy and Kurtz (2021), relational categories go beyond schemas in that they encompass not only abstract semantic information, but also knowledge about how the specific cases instantiate them. Building on this idea, they showed that fostering both the extraction of abstract semantic knowledge from specific cases and its application to new examples promotes structurally-based retrieval. Our results extend these findings by showing that highly familiar SGCs extracted through repeated analog experiences and routinely used to make sense of everyday situations are particularly likely to elicit the retrieval of distant analogs (Hofstadter & Sander, 2013). Thus, the high familiarity with some SGCs appears to support both activation of these categories when faced with their instances and retrieval of specific cases once the category is activated (Raynal & Sander, 2025).

The possibility that structurally-based retrieval depends on the availability of a familiar SGC also suggests an explanation for discrepant findings between previous studies investigating the retrieval of familiar events (e.g., popular movies in Trench & Minervino, 2015) and our study. Indeed, structurally-based retrieval may be more likely to occur when the stimuli match a single pre-existing SGC in memory (as in our study) than when they do not (e.g., as in Trench & Minervino, 2015). This possibility could be explored in future research investigating how the extent to which target cues match a pre-existing SGC affects structurally-based retrievals. Although structurally-based retrievals may be most frequent when structures match a single preformed SGC, there is evidence suggesting that they are also prone to occur when multiple familiar SGCs can be combined to encode novel structures (Raynal et al., 2020, 2024). Overall, we suggest that the generality of the dominance of surface similarities in analogical retrieval should be tempered, as it may only hold in cases where situations do not match a familiar schema from memory.

The present contribution also bears implications for the mechanisms underlying the retrieval of close analogs. Previous accounts have suggested that close analogs dominate over distant ones due to a surface-oriented retrieval mechanism that operates in a “kind” world where surface and structural similarities are generally aligned (Gentner & Medina, 1998). However, our findings indicate that, at least during SGC-mediated retrieval, the dominance of close analogs is explained by a retrieval process that is primarily driven by structural similarities, with surface features aiding in source selection (Raynal & Sander, 2025). Moreover, the combination of surface and structural similarities may drive retrieval most effectively by maximizing structural overlap between situations, as close analogs often preserve relevant structural features that distant analogs do not (Raynal et al., 2020). For example, two scenarios in which someone fails to maintain a fitness routine can both imply deviating from typical beauty standards, while failing to complete homework may lead to a different outcome. This “maximal structural overlap” hypothesis is indirectly supported by evidence that inferred relational features are automatically activated by objects (Bassok et al., 2008), and that inferred relations can serve as efficient retrieval cues (Popov et al., 2017).

## Generalizability and future directions

Our findings raise the question of the links between exemplar frequency and analogical retrieval. Prior research on categories has found that exemplar frequency − as estimated by word frequency (e.g., the number of occurrences of the word “pear” in books for the fruit category) − does not consistently correlate with production frequency in exemplar generation tasks (Mervis et al., 1976; Plant et al., 2011). However, the data from Experiment 3 showed that participants presented with labels of SGCs (e.g., *pretending to like a gift*) retrieved frequent instances (i.e., frequent surface/structure combos, e.g., pretending to like a cloth received as a gift) more often than less frequent ones (e.g., pretending to like a pen received as a gift). This apparent discrepancy may stem from the fact that our materials were constructed so that infrequent exemplars would be far more infrequent than those investigated in previous research on categories (e.g., *kumquat* for the fruit category). If infrequent exemplars are hardly available in participants’ memory, it is not surprising that frequency has a strong influence on analogical retrieval. Further, research on categories has generally found that typicality is a better predictor of exemplar production than word frequency (Mervis et al., 1976; Plant et al., 2011). Thus, a complementary explanation of our findings stemming from a category-based approach to analogical retrieval is that instances with usual surfaces may be more prototypical members of the critical SGC than unusual combos. Based on recent findings demonstrating prototypicality effects in relational retrieval (Popov et al., 2020), we suggest that future studies could further explore how the prototypicality of a source analog promotes the likelihood of its retrieval (Raynal & Sander, 2025).

A further issue emerging from our study concerns the types of SGCs that non-experts are most proficient at using to retrieve distant analogs. Research on familiar SGCs including the present one have mainly focused on categories characterizing social and everyday life scenarios involving human interactions (e.g., *robbery, superstition, making up an excuse*; Minervino et al., 2023; Olguín et al., 2022). It is plausible that participants are particularly familiar with such categories and especially prone to encoding the structure of their instances. There is also evidence that non-experts become able to generate distant analogies in other domains when they become sufficiently familiar with a relational category from this domain (e.g., *spatial arrangement*; Snoddy & Kurtz, 2024). Thus, we suggest that structurally-based retrievals are likely to occur in social domains due to the familiarity of many SGCs within these domains, but also extend to other domains where SGCs are sufficiently familiar.

The current study has documented the types of analogies individuals draw in everyday life with highly familiar SGCs. One might question if these analogies, based on familiar SGCs (e.g., *robbery*), constitute true analogies or simpler comparisons. Analogies relying on SGCs may sometimes be based on a single relation (e.g., x *steals* y), whereas traditional analogies involve interconnected relations (e.g., a planet *revolves* around the sun due to gravitational *attraction*; Gentner, 1983). However, as highlighted by Olguín et al. (2022), even though some familiar SGCs can be well captured by a single word, they may involve systems of relations rather than single relations (see Corral & Jones, 2026, for a similar argument). For instance, Olguín et al. (2022) have argued that “the SGC *robbery* tends to involve an intentional agent whose action results in something that belongs to the patient becoming the property of the agent, but without the consent of the patient” (p. 1402). Further, familiar SGC such as proverbs (e.g., *once bitten, twice shy*) can support the encoding of particularly complex relational patterns and retrieval based on whole structures (Hofstadter & Sander, 2013; Schank, 1999; Tullis & Goldstone, 2016). Accordingly, the analogies examined here fit the traditional definition, as they involved at least two interconnected relations. For example, memories were classified as *giving up a resolution* if they involved *making up a resolution* but *giving up on it*. Other materials used in previous studies exhibit similar relational complexity, as in stories like “Karla the Hawk” and its analog, which contain only two causally linked relations (i.e., *making a deal* causes *avoiding a threat*; Gentner et al., 1993). In contrast, the “Radiation problem” and its analogs feature a more complex structure with multiple goals and constraints (Gick & Holyoak, 1983). Thus, future research could investigate the complexity of everyday analogies based on SGCs and whether specific expertise facilitates the understanding of more complex ones.

Other differences in the stimuli used in the current study and in previous research are worth noting regarding the salience of surface and structural features in the target descriptions (Raynal & Sander, 2025). Our target cues were shorter than those used in traditional two-phase paradigms but similar in length to those used in other studies on naturalistic source retrieval (see Table 1). While the compactness of our materials may reproduce the format of anecdotes from everyday life interactions, future research could examine how our findings generalize to longer narratives that require condensing multiple events into a unified representation (Minervino & Trench, 2024). Additionally, the target cues in the current study involved two surface features (e.g., *painter, exhibition*), which is fewer than most studies that have resorted to the two-phase paradigm. In line with the empirical approach developed by Trench et al. (2025), this limited number was chosen to create fair competition between an equal number of object and relational cues (e.g., two surface features and two structural segments). Based on predictions from a retrieval mechanism initially driven by content similarity (as instantiated in MAC/FAC; Forbus et al., 1995), surface matches may become stronger competitors to distant analogs when situations involve a higher proportion of objects than relations. While this suggests that the dominance of surface matches is not necessarily due to object cues exerting a greater influence than relational cues in eliciting memory retrieval (see Popov et al., 2017, 2020, for a similar argument), further research is needed to determine how our findings apply to stimuli involving more surface similarities.

Lastly, the objects described by the target cues in Experiment 2 might be frequent in some cases (e.g., *pen, university*) but rarer in others (e.g., *knitting, garment*). The retrieval of surface matches may have been underestimated in cases where they were sparse in memory, and perhaps less personally significant than distant analogs. Conversely, surface cues may be less influential on retrieval when they involve commonplace objects, as suggested by research on episodic memory that has highlighted that the diagnostic value of a cue is a determinant for retrieval (Nairne, 2002; Poirier et al., 2012). Nevertheless, the structural superiority was observed for all target cues in the current study, suggesting that the dominance of structural similarities is not strictly dependent on the frequency of the surface cues.

## Conclusions

In sum, we have argued that humans are capable of using familiar SGCs to classify their incoming experiences and retrieve past situations based on structural similarities. In this view, the ability to retrieve purely structural matches has been previously underestimated in prior research investigating the role of SGC in analogical retrieval due to a focus on “kind world” settings in which surface and structural similarities are systematically correlated, offering participants the opportunity to retrieve close analogs. In line with this, we have demonstrated that dissociating these correlations by presenting participants with experimental stimuli instantiating familiar SGCs and whose structural and superficial features tended to cue contrasted memories led to massive structurally-based retrievals. Thus, assessing how analogical retrieval operates in a “misleading experimental world” where surface and structure do not correlate may have allowed us to better understand how it works in a kind world where surface and structural features are often associated.

## Data Availability

Data are available from the first author upon reasonable request.

## Footnotes

1 Note that although this phenomenon has not been explicitly related to blocking effects identified in episodic memory research, that give rise to a difficulty to generate a word (e.g., ALLERGY) given a word fragment (A_L_ _ GY) when primed with a related word (e.g., ANALOGY), they may involve common inhibitory mechanisms between source candidates sharing some kind of similarities (Schilling et al., 2014; Smith & Tindell, 1997; Thagard et al., 1990).

2 Note that because Experiment 1 was aimed at reproducing a widely replicated finding, the memories collected were coded by a single rater. However, the data obtained in Experiment 2, where the main contribution of the study is introduced, were coded by two judges according to a very similar coding scheme, and the high inter-rater agreement in Experiment 2 supports the validity of the scoring procedure.
